# Oxidative stress responses in biofilms

**DOI:** 10.1016/j.bioflm.2024.100203

**Published:** 2024-05-23

**Authors:** Waleska Stephanie da Cruz Nizer, Madison Elisabeth Adams, Kira Noelle Allison, Megan Catherine Montgomery, Hailey Mosher, Edana Cassol, Joerg Overhage

**Affiliations:** Department of Health Sciences, Carleton University, 1125 Colonel By Drive, Ottawa, K1S 5B6, ON, Canada

**Keywords:** Biofilms, Oxidative stress, Antimicrobial resistance, Reactive oxygen species, Reactive chlorine species, Hydrogen peroxide, Hypochlorous acid

## Abstract

Oxidizing agents are low-molecular-weight molecules that oxidize other substances by accepting electrons from them. They include reactive oxygen species (ROS), such as superoxide anions (O_2_^−^), hydrogen peroxide (H_2_O_2_), and hydroxyl radicals (HO^−^), and reactive chlorine species (RCS) including sodium hypochlorite (NaOCl) and its active ingredient hypochlorous acid (HOCl), and chloramines. Bacteria encounter oxidizing agents in many different environments and from diverse sources. Among them, they can be produced endogenously by aerobic respiration or exogenously by the use of disinfectants and cleaning agents, as well as by the mammalian immune system. Furthermore, human activities like industrial effluent pollution, agricultural runoff, and environmental activities like volcanic eruptions and photosynthesis are also sources of oxidants. Despite their antimicrobial effects, bacteria have developed many mechanisms to resist the damage caused by these toxic molecules. Previous research has demonstrated that growing as a biofilm particularly enhances bacterial survival against oxidizing agents. This review aims to summarize the current knowledge on the resistance mechanisms employed by bacterial biofilms against ROS and RCS, focussing on the most important mechanisms, including the formation of biofilms in response to oxidative stressors, the biofilm matrix as a protective barrier, the importance of detoxifying enzymes, and increased protection within multi-species biofilm communities. Understanding the complexity of bacterial responses against oxidative stress will provide valuable insights for potential therapeutic interventions and biofilm control strategies in diverse bacterial species.

## Highlights

• Oxidizing agents induce biofilm formation.
• Biofilm matrix as a protective barrier against ROS and RCS.
• Antioxidant enzymes increase survival of biofilms against oxidants.
• Multi-species biofilms promote oxidative stress resistance.

## 1 Introduction

Bacterial biofilms are becoming a prevailing area of microbial research as their ubiquitous presence and influence across artificial and natural environments become more understood. Their abundance and resistance mechanisms make them an important field of study, and they are now widely accepted as the predominant mode of microbial existence in most environments [1]. Biofilms exist as complex multicellular communities of microbes that are diverse in species composition and phenotypic function. These structures are commonly defined as groups of cellular aggregates attached (or not) to surfaces and/or other cells and are self-contained in a matrix of extracellular polymeric substances (EPS) [2,3], which is composed of various biomolecules such as polysaccharides, proteins, lipids, and extracellular DNA (eDNA) [4]. The properties of the matrix combined with the unique biofilm phenotype allow them to be found in many harsh and often changing environments [1].

Although biofilms have many beneficial applications, such as in wastewater treatment [[5], [6], [7]], in the removal of contaminants and pollutants from the environment [5,8], and the production of bioplastics [9], they represent a critical threat in medicine and food industries [10]. They are the primary cause of chronic infections [[11], [12], [13], [14]] and can impact food safety and quality as they are the sources of food contamination [[6], [7], [15]].

Growing as biofilms confers many advantages to bacterial cells since these structures act as a shield against physical and chemical challenges. The lack of understanding of biofilm dynamics and the application of planktonic knowledge-based solutions to biofilms can lead to suboptimal eradication methods and the rise of resistant species [16]. Elimination strategies are further complicated by interspecies interactions in biofilms where the proximity of microbial cells within biofilms creates the ideal environment for the exchange of resistance-encoding plasmids, resulting in increased antimicrobial tolerance [17,18]. Biofilms can also evade host immune responses, making biofilm colonization in human infections challenging to eradicate [19]. In healthcare settings, antibiotic misuse when treating biofilm infections has resulted in the global spread of antimicrobial resistance (AMR) and increased bacterial survival, which can lead to the development of life-threatening bacterial infections such as sepsis and bacteremia [20,21]. Moreover, their resilience against disinfectants and oxidizing agents commonly used in industrial, clinical, and domestic settings exacerbates the crisis created by AMR.

While extensive research has focused on investigating the oxidative stress responses to oxidizing agents in planktonic cells, the study of these responses employed in biofilms is still in its infancy, and only a few mechanisms have been reported. Therefore, this review aims to shed light on the current state of knowledge regarding the resistance mechanisms employed by bacterial biofilms to ROS and RCS, providing a comprehensive overview and stimulating further research in this crucial area of study.

## 2 Oxidizing agents

**Fig. 1:**
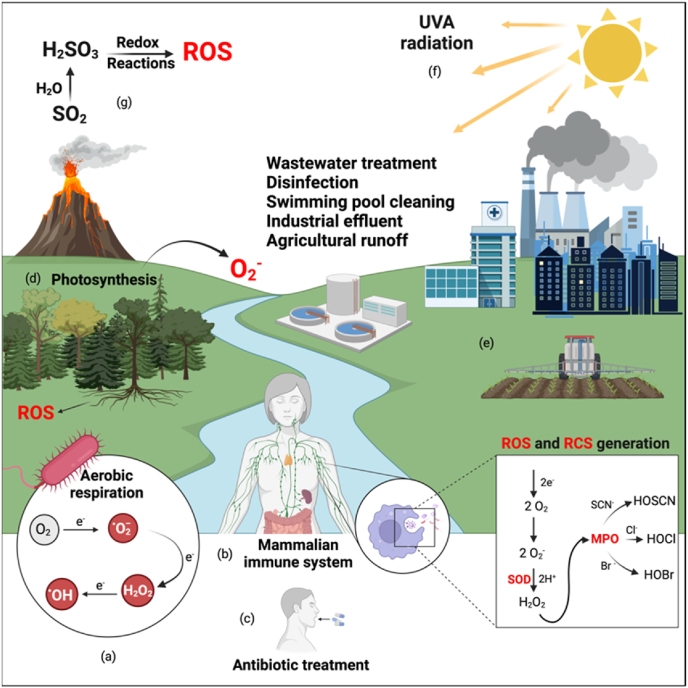
Sources of oxidative stress in bacteria and biofilms. Bacteria encounter toxic reactive species such as reactive oxygen and chlorine species (ROS and RCS, respectively) from diverse sources. (a) These toxic molecules can be endogenously produced by aerobic respiration, in which molecular oxygen (O_2_) acquires electrons and is converted into superoxide anions (O_2_^−^), hydrogen peroxide (H_2_O_2_), and hydroxyl radicals (HO^−^). (b) ROS and RCS can also be produced by the mammalian innate immune system during phagocytosis. (c) Some antibiotics, such as quinolones and β-lactams, are also known to induce oxidative stress in bacterial cells through many different mechanisms. (d) Plants can also generate toxic oxygen species during photosynthesis or by their roots as, for example, a response to stress. (e) Human activities, including agriculture and the release of industrial effluent pollution, are also important sources of oxidants. Furthermore, disinfectants and cleaning agents such as bleach in wastewater treatment, as well as in industrial, domestic, and hospital settings, are also important sources of ROS and RCS. On a bigger scale, (f) UVA radiation and (g) volcanic gases can also generate toxic reactive species. Cl-, Br^−^, and SCN^−^: chloride, bromide, and thiocyanate anions, respectively; HOCl: hypochlorous acid; HOBr: hypobromous acid; HOSCN: hypothiocyanite; SOD: superoxide dismutase; MPO: myeloperoxidase. Created with BioRender.com.

Oxidizing agents or oxidants are low-molecular-weight molecules that oxidize other substances by accepting electrons from them. These molecules possess high reactivity due to the presence of unpaired electrons, a high degree of electron deficiency, or other destabilizing characteristics and can be generated from various natural and anthropogenic sources. The intracellular production or accumulation of oxidizing agents leads to oxidative stress [22]. The most biologically abundant and clinically relevant reactive molecules include reactive oxygen, chlorine, nitrogen, and sulphur species (ROS, RCS, RNS, and RSS, respectively). While the oxidant effect and oxidative stress responses of RNS and RSS have been reviewed previously [23,24], the focus of this review is on ROS and RCS. These oxidants disrupt the redox balance within bacteria, eventually precipitating an intracellular state wherein oxidant generation exceeds the bacterial detoxification and repair capacities, thus triggering irreversible cell damage and eventual death (reviewed in [25]. Furthermore, oxidative stress can cause damage to DNA, proteins, and lipids. These lesions can lead to mutations if they are not adequately repaired before DNA replication. In this context, ROS-induced DNA damage is of particular significance since it causes base modifications, the formation of mismatched base pairs, and DNA strand breaks [26,27]. In some cases, oxidative-induced mutagenesis can lead to the acquisition of mutations that confer resistance to antibiotics or other environmental stresses, allowing bacteria to survive and proliferate in hostile conditions [28,29]. Since bacteria and bacterial biofilms are ubiquitous in the most diverse environments, they are continuously exposed to oxidizing agents from the most diverse sources, as illustrated in Fig. 1.

Molecular oxygen (O_2_), while essential for aerobic life, is a primary source of endogenous oxidant generation in bacteria due to its tendency for partial reduction reactions during cellular aerobic respiration, which subsequently induces the formation of ROS, including superoxide anions (O_2_^−^), hydrogen peroxide (H_2_O_2_), and hydroxyl radicals (HO^−^) [30,31]. Additionally, some bacterial species may maliciously produce and secrete ROS as a survival advantage to thrive in environments rich in microbial diversity, ultimately securing themselves metabolic resources and physical space by suppressing the growth of competing microorganisms [32]. Other environmental factors capable of inducing bacterial ROS production include pH and temperature extremes [33], nutritional deprivation [34], and sunlight irradiation [35]. Among them, ultraviolet radiation A (UVA) generates intracellular ROS in bacteria by different mechanisms. For instance, it interferes with electron transport chain (ETC) components present in bacterial cell membranes, which can lead to electron leakage from the ETC and the generation of ROS [36]. Furthermore, when exposed to UVA, photosensitizers, such as porphyrins, can transition to higher energy states, becoming electronically excited, which results in the transfer of energy to neighboring molecules, including O_2_ present in the environment [37]. ROS generation by UVA and the damage caused by UVA to DNA and RNA are also considered the primary mechanisms of cell-associated killing [35,[38], [39], [40]].

The mammalian innate immune system employs a sophisticated and powerful arsenal against pathogenic bacteria, with reactive species, particularly ROS and RCS, being key components of this defence mechanism. Upon pathogen encounter, phagocytes, such as neutrophils and macrophages, undergo an oxidative burst, in which they rapidly increase O_2_ consumption to produce ROS, a process driven by NADPH oxidase. NADPH oxidase is assembled and activated in phagosomal membranes to catalyze the conversion of O_2_ into O_2_^−^. O_2_^−^ can be further converted into other ROS, such as H_2_O_2_ and HO^−^ [41]. Plants also protect themselves from pathogenic bacterial colonization through ROS production using similar mechanisms and pathways [[42], [43], [44]]. In innate immune cells, myeloperoxidase (MPO) uses H_2_O_2_ and chloride ions (Cl^−^) to produce hypochlorous acid (HOCl), a potent RCS with arguably stronger and faster bactericidal action than ROS. It is estimated that 90 % of O_2_^−^ is converted into HOCl [45]. HOCl further reacts with phagosomal amines to form chloramines, another potent RCS [45,46]. Furthermore, ROS can also serve as signaling molecules driving immune responses, with mitochondrial ROS, for example, influencing cytokine production and eliciting inflammatory responses [47].

Due to their potent antimicrobial properties and broad-spectrum effectiveness by the oxidation of various cellular components, ROS and RCS are critical molecular components of various cleaning and disinfection agents. For example, HOCl constitutes the active ingredient in sodium hypochlorite (NaOCl), commercial bleach, and arguably the most widely utilized disinfectant in domestic, clinical, and industrial contexts [48]. Moreover, various formulations of reactive species are utilized in wastewater treatment and sanitation [49], medical and dental applications [50,51], swimming water chlorination [52], and beyond.

Exposure to antibiotics also induces oxidative stress in bacterial cells. In this context, the relationship between antibiotics and oxidative stress is a topic of active research (reviewed in [53,54]). Indeed, certain antibiotics, such as quinolones and β-lactams, are commonly associated with the induction of oxidative stress in bacteria [55]. For example, quinolones can generate O_2_^−^ and OH^−^ radicals by inhibiting the bacterial DNA gyrase and promoting the Fenton reaction [[56], [57], [58]]. The killing effect of the quinolone ciprofloxacin was also shown to be enhanced by the production of OH^−^ radicals in *P. aeruginosa* biofilms [59].

Moving toward the bigger picture of environmental-level bacterial exposure to oxidants, oxidizing agents in soil and water originate from various biotic and abiotic processes, as ROS play significant roles in soil chemistry while also affecting plant-microbe interactions. During metabolic activities like photosynthesis, organic matter decomposition, and nitrogen fixation, both terrestrial and aquatic plants, fungi, and phytoplankton produce reactive species as by-products [[60], [61], [62]]. For example, the light-driven electron transport in chloroplasts can lead to the formation of O_2_^−^ and, subsequently, other ROS [63]. Furthermore, plant roots can release ROS into the soil environment as a stress response or as a mechanism to facilitate nutrient uptake [64]. Other natural geological processes, such as volcanic eruptions, also release compounds into soil and water bodies, contributing to oxidant generation. For instance, sulphur dioxide (SO_2_) from volcanic gases can dissolve in water to form sulphurous acid, which can participate in redox reactions generating ROS [65]. Beyond natural reactive species-generating phenomena, human activities such as industrial effluent pollution and agricultural runoff can induce contaminants into the natural environment that promote oxidant generation, thus modifying the composition and metabolic functions of microbial populations [66]. Environmental and anthropogenic sources of ROS can eventually result in AMR by, among other mechanisms, inducing mutations [67].

### 2.1 ROS and RCS as treatment options

AMR pathogens are consistently found in hospital settings and are believed to be one of the biggest causes of nosocomial infections, putting those with chronic conditions, such as chronic wound patients, at a greater risk of carrying and circulating resistant infections [68]. Due to the increase in these resistant strains in healthcare settings, the development of antibiotic alternatives to kill nosocomial strains is on the rise. Unlike antibiotics, which often act on specific microbial processes to affect certain bacterial mechanisms [69], oxidizing agents possess a broader mode of action. They are less specific, as their bactericidal mechanisms include the non-specific disruption of cellular membranes or interaction with organisms’ functional groups [70]. Among the many applications of oxidizing agents is their use as treatment options, such as in wound care.

Investigations into the use of H_2_O_2_ as a disinfectant in chronic tissue infections found that H_2_O_2_-treated gauze increases skin graft uptake in chronically infected burn wounds [71]. Ointments containing H_2_O_2_ [72] and wound dressings containing glucose oxidase incorporated into a collagen matrix [73] increase angiogenesis and improve overall wound outcomes in *in vivo* models. In a review by Dunnill et al. [74], the authors outlined how ROS can provide new therapies for wound healing that go beyond a disinfectant. Several strategies are discussed, including employing topical ROS or their intermediates as antibacterial agents and enhancers of angiogenesis, the use of ROS-generating glucose oxidase, hyperbaric O_2_ therapy to alleviate wound hypoxia and bolster phagocytic respiratory burst efficiency, and the application of galvanic particles to reduce inflammation and promote the ROS-mediated fibroblast migration [74]. Other novel treatments centered on ROS modulation and ROS scavenging analogs have utilized antioxidative enzymes and sprayable hydrogels containing ROS nanoparticles and antimicrobial peptides to promote wound healing [75,76]. However, other studies have found that H_2_O_2_ treatment can impair wound healing [77] and has little impact on increasing cell proliferation in the healing stages [78]. Furthermore, high levels of ROS exert harmful effects on host cells by directly impacting proteins, lipids, and nucleic acids, ultimately leading to cell death through several mechanisms, such as necroptosis and apoptosis [79,80].

Another oxidizing agent that has been used in health care settings both in tissue and surface disinfection is HOCl, which acts against almost all viral, bacterial, and fungal pathogens. Multiple studies have found that HOCl has potent antimicrobial and antibiofilm effects on various microorganisms as a liquid disinfectant and topical ointments/gels for biofilm-infected chronic wounds [81,82]. In similar cases, HOCl antibacterial activity was complemented by a 70–90 % reduction in EPS [83] and a dose-dependent positive impact on the migration of fibroblasts and keratinocytes [84]. However, as observed for ROS, harm to the host remains a concern, as studies have found that increased HOCl cytotoxic effects coincide with increased antimicrobial effects [85]. These studies suggest that HOCl could aid in addressing tissue biofilm infections by reducing bacterial load and penetrating the EPS. The use of oxidizing agents in health care interventions requires a balance to maintain homeostasis, enough to promote healing without inflicting damage to the host.

## 3 Oxidative stress resistance in bacterial biofilms

Despite the constant exposure and the potent antimicrobial and antibiofilm effects exerted by oxidizing agents, bacteria have developed many strategies to survive the cellular damage caused by these toxic substances. In this section, we summarize the main stress resistance mechanisms described for bacterial biofilms against ROS and RCS.

### 3.1 Biofilm formation as a response to oxidative stress

**Fig. 2:**
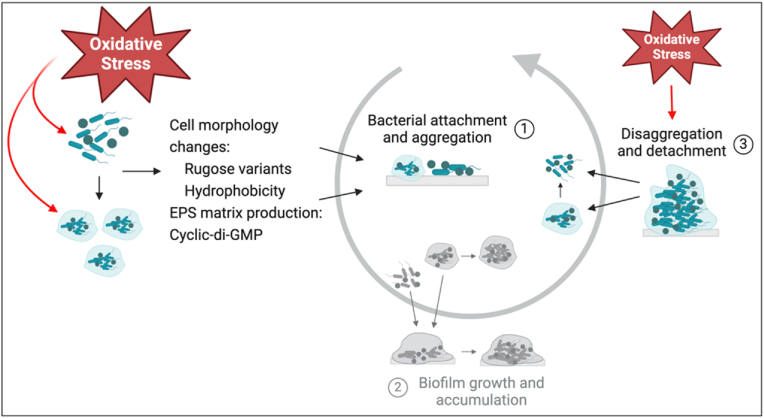
Stimulation of biofilm formation by oxidative stress. Oxidative stress induces cell adhesion by inducing changes in cell morphology by, for example, the development of rugose variants and an increase in cell hydrophobicity. Furthermore, these toxic species increase the production of the EPS matrix and its components as well as cyclic-di-GMP. These processes induce the initial step of biofilm development (step 1). Additionally, oxidants such as NaOCl disrupt the biofilm matrix, promoting the release of single cells or cell aggregates, which can then colonize other sites (step 3). Biofilm formation process described by the three-step inclusive model proposed by Sauer et al. [2]. Created with BioRender.com.

Although oxidizing agents can disrupt biofilms [86,87], they can also promote [88,89] biofilm formation as a protective mechanism against these toxic substances [90]. However, many mechanisms driving this process are yet to be fully understood. Fig. 2 illustrates the mechanisms involved in biofilm formation induced by oxidative stress.

Oxidizing agents are known to induce changes in cell morphology, which is a factor that significantly influences biofilm formation. In this context, sublethal concentrations of NaOCl induced the formation of a rugose variant in *Salmonella* Heidelberg. This change in cell morphology was accompanied by the formation of aggregates and robust biofilms and increased resistance to chlorine [91]. In *Escherichia coli*, intracellular ROS accumulation triggered the development of rugose biofilms upon iron exposure, which displayed increased H_2_O_2_ resistance compared to rugose biofilms grown in low-iron conditions [92].

Additionally, the strong biofilm formation capability of *E. coli* cells previously exposed to increasing sub-inhibitory concentrations of NaOCl was associated with increased cell hydrophobicity and the development of elongated, rougher, and deformed cells. These NaOCl-adapted cells also presented resistance to high doses of NaOCl [88].

Considering the protective effect of the EPS matrix [93,94] and its importance in biofilm attachment [95], it is not surprising that inducing the production of this crucial biofilm structure and its components is a mechanism employed by bacterial cells in response to the damage caused by oxidizing agents. In this context, increased EPS production by the Antarctic bacterium *P. extremaustralis* upon exposure to H_2_O_2_ was correlated with a 7.5-fold increase in the expression of *pgaD* involved in the synthesis of the protein dPNAG, which plays roles in cell-cell and cell-abiotic surface adhesion [96]. Enhanced EPS production by the upregulation of the PNAG operon and increased biofilm formation was also found in an alkyl hydroperoxide reductase (*ahp*) *Acinetobacter oleivorans* mutant [89]. The depletion of the EPS matrix by NaOCl and the consequent formation of cell clusters can also contribute to the spread of planktonic cells or aggregates and the formation of biofilms in new sites [97]. Moreover, the deletion of the central oxidative stress regulator OxyR in *P. chlororaphis* caused increased biofilm production as the mechanism to resist oxidative stress [98]. These findings suggest that biofilm formation, EPS matrix production, and other well-known oxidative resistance mechanisms are tightly regulated in cells experiencing stress.

In many bacteria, biofilm formation is mediated by the second messenger cyclic-di-GMP (c-di-GMP), which controls many cellular mechanisms, including matrix production [99]. A sub-lethal concentration of NaOCl was shown to induce a 26-fold increase in the expression of PA3177, a diguanylate cyclase that catalyzes the synthesis of c-di-GMP [100]. In another study, *P. aeruginosa* PA3177 mutant biofilms presented a 3-log reduction in the number of cells after treatment with H_2_O_2_ as opposed to a 0.6-log reduction in the WT biofilms, while overproduction of PA3177 restored the susceptibility phenotype in the mutant strain [101].

Evidence suggests that *P. aeruginosa* biofilm tolerance is likely linked to early biofilm development and induced upon transition to the irreversible attachment stage [102]. However, the intricate interplay between oxidative stress and biofilm formation underscores the complexity of bacterial responses to environmental challenges; in which understanding the complexity of bacterial responses to oxidative stress will provide valuable insights for potential therapeutic interventions and biofilm control strategies in diverse bacterial species.

### 3.2 Biofilm matrix: a protective barrier against ROS and RCS

In addition to its involvement in the overall stability and functionality of biofilms [103], the EPS matrix has been shown to increase the resistance of these structures to diverse oxidizing agents such as NaOCl [104], H_2_O_2_ [105], and calcium peroxide (CaO_2_) [106]. The main matrix components associated with oxidative stress resistance in biofilms are summarized in Table 1.

**Table 1:** Summary of matrix components previously shown to be involved in the resistance of bacterial biofilms to ROS and RCS.

Matrix component	Bacterial species	Oxidizing agent	Reference
**eDNA**	*Haemophilus influenzae*	NaOCl	[107]
	*S. epidermidis*	NaOCl and H_2_O_2_	[108]
**Alginate**	*P. aeruginosa*	H_2_O_2_	[109]
	*P. aeruginosa*	H_2_O_2_	[110]
	*P. aeruginosa*	H_2_O_2_	[111]
	*P. aeruginosa*	UVA	[112]
	*P. aeruginosa*	NaOCl	[113]
**Psl and Pel**	*P. aeruginosa*	UVA, NaOCl, and H_2_O_2_	[114]
**Linear homo-polysaccharides**^a^	*Lactococcus hircilactis* and *Lactobacillus delbrueckii*	H_2_O_2_	[115]
**NS**^b^	*Bacillus cereus*	Chlorine ions from electrolyzed water	[116]
**NS**^b^	*Klebsiella* H1*, Pseudomonas* C5*, Flavobacterium* GS3*,* and *Sphingomonas* Z22	NaOCl	[97]

a Polysaccharide names not provided.

b NS:Matrix component not specified.

One important aspect of the matrix that contributes to the increased resistance of biofilms to antimicrobials, including oxidizing agents, is that this structure slows down the diffusion of these agents into the biofilm [[117], [118], [119]]. In this context, non-typeable *Haemophilus influenzae* (NTHi) biofilms presented increased resistance to NaOCl due to eDNA, the primary component of the biofilm matrix [107]. The increased release of eDNA by *Staphylococcus epidermidis* biofilms exposed to NaOCl or H_2_O_2_ compared to untreated control biofilms and planktonic cells reinforces the role of eDNA in biofilm resistance to oxidants [108]. In this study, two hypotheses were proposed to elucidate the increased release of eDNA by biofilms compared to planktonic cells. First, a highly regulated process involving factors such as the protection of eDNA against DNase degradation, binding of eDNA to EPS components, reduced DNase production by biofilms, and the release of enzymes that inactivate DNase was proposed. Second, oxidizing agents would be neutralized by the increased production of biofilm matrix and catalase [108].

The low antimicrobial penetration into the matrix is vastly attributed to the reaction of EPS matrix components with oxidizing agents. Since chlorine ions in electrolyzed water with high oxidation-reduction potential have been previously shown to react with EPS components, the increased resistance of *Bacillus cereus* biofilms to this antimicrobial agent was attributed to the overproduction of EPS matrix and high biofilm biomass [116]. Additionally, the detection of cell clusters after the treatment of *Klebsiella* H1, *Pseudomonas* C5, *Flavobacterium* GS3, and *Sphingomonas* Z22 biofilms with chlorine indicates that the reaction of NaOCl with the EPS matrix might lead to the depletion of the biofilm matrix, provoking the formation of small cell clusters [97].

Alginate, a matrix polysaccharide known to react with oxidants, presents a scavenging function and decreases the killing effect of these molecules [113,120]. It was previously shown that the uronic acid core and the O-acetyl groups of *P. aeruginosa* alginate react with NaOCl and quench its toxic effect [113]. In *P. aeruginosa*, the overproduction of alginate is characteristic of the mucoid phenotype, which has been shown to increase the resistance of this bacterium to polymorphonuclear leukocytes (PMNs) and oxidizing agents such as H_2_O_2_ [109]. Furthermore, sub-lethal concentrations of H_2_O_2_ induced the formation of mucoid *P. aeruginosa* variants with 2- to 6-fold higher alginate quantities compared to wild-type biofilms, leading the authors to suggest that alginate overproduction serves as a bacterial defence system against immune-derived oxidants like H_2_O_2_ [110].

The absence of genes involved in the alginate biosynthetic pathway has also been shown to alter the susceptibility of biofilms to oxidants, corroborating the roles of alginate production in biofilm resistance. For instance, *algT* mutant biofilms presented increased susceptibility to H_2_O_2_ than wild-type biofilms [111]. On the other hand, the addition of alginate to *P. aeruginosa* PAO1 wild-type and Δ*algD* mutant strain biofilms increased their resistance to UVA radiation, a source of intracellular ROS formation [112].

Besides alginate, the other polysaccharides produced by *P. aeruginosa* (i.e., Psl and Pel) were also shown to play important roles in the resistance of PAO1 biofilms to UVA, H_2_O_2_, and NaOCl, with Pel playing important roles in oxidative stress resistance [114]. Moreover, exopolysaccharides isolated from *Lactococcus hircilactis* and *Lactobacillus delbrueckii* presented an H_2_O_2_-scavenging effect due to the presence of ribose groups on their structures [115].

In addition to conferring slow penetration of oxidizing agents, the EPS matrix also provides enough time for the bacteria to adapt and activate other resistance mechanisms [121], representing a physical and chemical barrier of biofilms. While the EPS matrix is a crucial determinant in biofilm resistance, it alone cannot entirely account for this phenomenon [108,122,123]. In this sense, the resistance of biofilms to antimicrobial agents is multifaceted, involving various mechanisms, such as the presence of detoxifying enzymes.

### 3.3 Detoxifying enzymes

Current research suggests that bacterial biofilms’ remarkable resilience against oxidizing agents may be achieved, at least partially, by detoxifying enzymes like catalases, Ahp, and superoxide dismutases (SODs) [[106], [124], [125]]. These protective proteins mediate the degradation of endogenous and exogenous ROS and other oxidizing agents [126]. This adaptive strategy bears a resemblance to the protective measures employed by planktonic cells challenged by oxidative stress [[127], [128], [129], [130]], indicating a conserved mechanism of stress response across different bacterial lifestyles.

The H_2_O_2_ responsive transcriptional regulator OxyR, the so-called master regulator of oxidative stress, triggers the expression of numerous antioxidant defense genes, including *katA*, *katB*, *ahpB*, and *ahpCF* [131], mostly during the exponential growth phase. Interestingly, the impact of OxyR on biofilm resistance to peroxides appears relatively minor [132]. Other global regulators that control the expression of antioxidant enzymes and, consequently, the survival of bacterial biofilms under oxidative stress include the Las and Rhl quorum-sensing system, regulating catalase and SOD expression [133] and the sigma factor RpoS [111,132], to which *katE* expression is predominantly dependent [132].

In the context of studying specific antioxidant enzymes within biofilms and their corresponding biochemical pathways and regulatory networks concerning oxidative stress, bacterial catalases (namely *katA, katB, katC/katE,* and *katG)* constitute the most studied group [105,123,125,132,[134], [135], [136], [137], [138]]. They are critical to breaking down H_2_O_2_ into H_2_O and O_2_. However, the relative catalase activity in wild-type biofilms compared to planktonic cells remains unclear. Studies conflictingly report that *P. aeruginosa* biofilms exhibit lower catalase activity upon oxidant treatment [125,133], *S. epidermidis* possess greater catalase levels in biofilm states [137], and similar catalase expression patterns in both modes of growth were described for *P. aeruginosa* [123,125]. Indeed, catalase levels within oxidant-exposed biofilms depend on biofilm age [132] and species-specific catalase metastability and cellular localization [138]. Overall, however, catalase-deficient biofilms are generally reported to possess increased susceptibility to oxidizing agents, particularly H_2_O_2_, compared to their wild-type biofilm counterparts [133,136,138]. In terms of catalases' specific mechanisms of protection in biofilms, Zhang et al. [121] recently showed that *E. coli* biofilms utilize two patterns of H_2_O_2_ resistance. First, catalases act as a barrier to block the penetration of H_2_O_2_ into the biofilms. Second, considering that O_2_ is a growth-limiting factor, the breakdown of H_2_O_2_ and the production of O_2_ promotes biofilm growth since O_2_'s cross-membrane rate is faster than that of H_2_O_2_ [121].

Other detoxifying enzymes have also been examined in relation to biofilm reactions to oxidizing agents; however, the literature on this subject is sparse, with only a handful of isolated studies delving into their roles. SODs catalyze the dismutation of O_2_^−^ into O_2_ and H_2_O_2_, which is less toxic than O_2_^−^. H_2_O_2_ is then further broken down into water and oxygen by other antioxidant enzymes like catalases and peroxidases, completing the detoxification process. *E. coli sodC* mutant biofilms were more susceptible to H_2_O_2_ treatment than the wild-type strain [139]. Furthermore, SODs appear to play a major role in population-level bacterial stress responses to calcium peroxide [106]. Another antioxidant defence is Ahp, which reduces hydroperoxides, including H_2_O_2_ and organic hydroperoxides (ROOH), to their corresponding alcohols (ROH) and H_2_O. Interestingly, *P. aeruginosa ahpCF* mutant biofilms were more susceptible to H_2_O_2_ than *katB* mutant biofilms treated with this disinfectant [125]. Moreover, *E. coli* O157:H7 *ahpC* displayed protective effects in 48-h biofilms [132].

The use of antioxidant enzymes as a resistance mechanism employed by different bacterial species is summarized in Table 2. Evidence suggests that antioxidant enzymes cannot fully explain the increased resistance of biofilms to oxidizing agents compared to planktonic cells. For example, although blue light photoinactivation of catalase within *P. aeruginosa* biofilms rendered these sessile populations more susceptible to H_2_O_2_ treatment, microbial biofilms were not completely eradicated using this blue light-H_2_O_2_ synergistic treatment, even at the highest H_2_O_2_ concentration [122]. Additionally, a *rhII* mutant strain showed wild-type-like catalase activity yet was significantly more susceptible to H_2_O_2_ treatment as compared to the wild-type strain [133]. Although *B. subtilis ahpA* was shown to be specifically expressed during biofilm formation, *ahpA* mutants could still form robust biofilms and resist peroxide treatment [140]. Taken together, these findings indicate that the resistance of biofilms to oxidative stress is a complex, dynamic trait involving multiple mechanisms beyond antioxidant enzyme activity alone, warranting further investigation into alternative pathways and strategies to further unravel oxidative stress responses of bacterial biofilms.

**Table 2:** Summary of studies reporting the increase in the resistance of bacterial biofilms to ROS and RCS due to antioxidant enzymes.

Antioxidant enzyme	Bacterial species	Oxidizing agent	Reference
**Catalase**	*P. aeruginosa*	H_2_O_2_	[133]
	*E. coli*	H_2_O_2_	[132]
	*P. aeruginosa*	H_2_O_2_	[125]
	*P. aeruginosa*	H_2_O_2_	[138]
	Nontypeable *H. influenzae*	H_2_O_2_ and neutrophil extracellular traps	[136]
	*E. coli*	H_2_O_2_	[121]
	*P. aeruginosa*	2,4-Dichlorophenoxyacetic acid	[124]
	*S. epidermidis*	NaOCl and H_2_O_2_	[137]
	*Salmonella enterica serovar Typhi*	H_2_O_2_	[105]
	*Vibrio fischeri*	H_2_O_2_	[134]
**SOD**	*E. coli*	H_2_O_2_	[139]
	*P. aeruginosa*	H_2_O_2_	[133]
	Sludge anaerobic bacteria	Calcium peroxide	[106]
**Ahp**	*P. aeruginosa*	H_2_O_2_	[125]
	*E. coli*	H_2_O_2_	[132]
	*P. aeruginosa*	2,4-Dichlorophenoxyacetic acid	[124]

### 3.4 Multi-species biofilms promote oxidative stress resistance

Although multi-species biofilms are the dominant form of biofilm in nature, much of the research concerning biofilms considered only single-species models [[141], [142], [143]]. However, growing as multi-species biofilms confers many advantages to the biofilm-dwelling organisms, such as protection against antimicrobial agents [144]. Table 3 summarizes the effect of multi-species biofilms as survival mechanisms against oxidative stress. For instance, when grown in a dual-species biofilm with *Veillonella parvula*, *S. mutans* demonstrated improved survival against H_2_O_2_ and amine chloride exposure compared to single-species biofilms [145]. Moreover, NaOCl presented a decreased antimicrobial effect against the dual-species biofilms of *P. aeruginosa* and the foodborne pathogen *Listeria monocytogenes* [146]. This resistance phenotype has also been observed for inter-kingdom populations. For example, biofilms formed by the filamentous fungi *Penicillium expansum* or *P. brevicompactum* and the bacterium *A. calcoaceticus* isolated from a drinking water distribution system presented lower biomass removal and inactivation than mono-species biofilms upon chlorination by NaOCl [147]. The positive interaction and protection in multi-species biofilms showed that *Aggregatibacter actinomycetemcomitans,* when grown in co-culture with *S. gordonii,* presents increased resistance to H_2_O_2_ due to the stimulation of the production of catalase KatA and the outer-membrane protein ApiA by *A. actinomycetemcomitans* in response to the H_2_O_2_ produced by *S. gordonii* [148]. Similarly, *S. mutans* and *L. casei* dual-species biofilms presented decreased resistance to H_2_O_2_, in which the upregulation of oxidative stress genes such as *sodA*, *nox1*, and *tp* in *S. mutans* was detected, suggesting that *L. casei* induces strong oxidative stress in this bacterium [149]. An *in vitro* analysis of an H_2_O_2_-producing e-bandage decreased viable cells in mono- and dual-species biofilms composed of Gram-positive and Gram-negative bacteria and was, therefore, suggested as a new option for treating chronic wounds [150].

**Table 3:** Impact of multi-species biofilms on the resistance to ROS and RCS.

Biofilm composition	Oxidizing agent	Effect^a^	Mechanism	Reference
*Veillonella parvula* and *S. mutans*	H_2_O_2_ and amine chloride	+ for *S. mutans*	Not described	[145]
*P. aeruginosa* and *Listeria monocytogenes*	NaOCl	+	Not described	[146]
*A. calcoaceticus* with *Penicillium expansum* or *P. brevicompactum*	NaOCl	+	Not described	[147]
*Aggregatibacter actinomycetemcomitans* and *S. gordonii*	H_2_O_2_	+ for *A. actinomycetemcomitans*	Induction of catalase and ApiA production by *A. actinomycetemcomitans* induced by *S. gordonii*	[148].
*Salmonella* wild-type with EPS or catalase mutant strains	H_2_O_2_	+	Production of colanic acid, Vi antigen, and catalase by the wild-type strain	[105]
*Fusobacterium nucleatum* and *Peptostreptococcus micros*	NaOCl	+	Increased biofilm biomass and biofilm matrix production	[151]
*E. coli* O157:H7 with *Bacillus* or *Acinetobacter*	NaOCl	+ for *E. coli*	Biofilm diversity and morphology	[152]
*E. coli* O157:H7 with other *E. coli* serotypes	H_2_O_2_	+ for *E. coli* O157:H7	Biofilm composition	[153]
*E. coli* with *Citrobacter*	H_2_O_2_	- for both strains	Biofilm composition	[153]
*E. faecalis* with *S. sanguinis* or *F. nucleatum*	NaOCl	- for *E. faecalis*	Nutrient, space, and adherence competition	[154]
*S. aureus* and *Salmonella* spp.	NaOCl, cetrimonium bromide, and peracetic acid	–	Not described	[155]
*S. mutans* and *L. casei*	H_2_O_2_	+ for both	Upregulation of oxidative stress genes such as *sodA*, *nox1*, and *tp* in *S. mutans*	[149]

a Multi-species biofilms increased resistance (+) or decreased resistance (−) to oxidants.

In many cases, a product synthesized by one species protects the entire biofilm population. For example, the EPS components colanic acid and Vi antigen and catalase produced by *Salmonella* wild-type biofilms protected the respective mutant strains when grown as multi-species biofilms with the wild-type strain. In this context, the biofilms became resistant to H_2_O_2_ due to EPS slowing its penetration and catalases regulating H_2_O_2_ levels [105]. The increased resistance of biofilms formed by *Fusobacterium nucleatum* and *Peptostreptococcus micros* was also attributed to the increased biomass and biofilm matrix production [151], reinforcing the importance of the EPS matrix in the protection of single species as well as multi-species populations.

A critical determinant when studying multi-species biofilms is the high diversity of these structures. Although *Bacillus* and *Acinetobacter* protected *E. coli* O157:H7 against the toxic effects of NaOCl, greater protection was seen in *Acinetobacter*-mixed biofilms [152]. Furthermore, *E. coli* O157:H7 co-cultured with other *E. coli* serotypes presented increased H_2_O_2_ resistance compared to planktonic cells. However, in *E. coli*-*Citrobacter* biofilms, both strains were deactivated by H_2_O_2_ [153]. These data show that biofilm diversity and function depend on the strains present in the biofilm, and resistance to oxidizing agents may be strongly influenced by the companion strain [152,153].

In addition to a positive interaction between the species in a biofilm, microbes grown as a consortium can also be negatively affected by each other. The analysis of biofilms formed by *Enterococcus faecalis* and *S. sanguinis* or *F. nucleatum* revealed that growing as multi-species biofilms did not confer any advantage to *E. faecalis* against NaOCl, possibly due to competition for nutrients, space, and adherence in the multi-species biofilms [154]. Similarly, biofilms of *S. aureus* and *Salmonella* spp. were more sensitive to NaOCl, cetrimonium bromide, and peracetic acid than mono-species biofilms [155].

While the advantages of multi-species biofilms are evident in their resilience and protective mechanisms, studying resistance to oxidants poses considerable challenges. The variability in the protective effects among biofilms formed by different strains underscores the complexities in unraveling resistance mechanisms within diverse multi-species biofilms. Understanding these challenges is vital to unravel the intricate interplay between biofilm phenotypes and their response to oxidative stresses. It will provide valuable insights for developing effective strategies in diverse applications, from healthcare to environmental management. Furthermore, understanding the mechanisms behind the resistance of multi-species biofilms to antimicrobials is especially important within a clinical setting where nosocomial infections can be obtained through the persistence of biofilms on medical devices.

### 3.5 Differential gene expression in biofilms in response to oxidative stress

Although less common than in planktonic cells, studies also conducted transcriptomic analyses to determine gene expression in biofilm-dwelling cells, shedding light on new molecules and pathways involved in resistance as well as confirming mechanisms previously identified. DNA microarrays found that *E. coli* YcfR, an outer membrane protein, was induced by several stress conditions, including H_2_O_2_ stress, suggesting it acts as a stress resistance molecule. Further analyses showed that *ycfR* reduces cell surface characteristics like hydrophobicity, aggregation, and biofilm formation [156].

Lipus and colleagues (2019) treated *P. fluorescens* biofilms with NaOCl and used RNA-sequencing to show that the most upregulated genes are involved in oxidative stress response (e.g., *ohr, ahpC, ahpF, trxB, yedY,* and *katA*), multidrug efflux, transport, and membrane related genes (e.g., *mexE, terC, ssuF, copZ, potAB, kdpA,* and *araJ*), and transcription regulation (e.g., *araC, tetR, lysR, iscR,* and *arsR*), reinforcing the roles of detoxifying enzymes in oxidative stress response in biofilms. These results suggest that *P. fluorescens* biofilms export NaOCl or its toxic by-products by upregulating efflux pumps like MexEF-OprN and other major facilitator superfamily (MFS) transporters [157]. In addition to the upregulation of genes involved in oxidative stress protection (e.g., *ahpC*, *ahpF*, catalase, thioredoxin reductase (*trxB* and *trxA*), *ohr*, and OsmC-like protein), the importance of catalase *katB* and the DNA repair system *recA* for the survival of *Burkholderia cenocepacia* biofilms to high levels of H_2_O_2_ and NaOCl was highlighted. Among the other genes upregulated are the iron-sulphur cluster (*iscSUA-hscAB-fdx-iscX* and *iscR*), major stress response sigma factors [RNA polymerase σ^32^ factor (BCAL0787), an ECF (extracytoplasmic function) σ^70^ factor OrbS (BCAL1688), a putative σ factor (BCAL3478) and an ECF σ^70^ factor FecI (BCAL1369)], and flagella-related genes [158]. Another study showed that *S. epidermidis* biofilms exposed to NaOCl or H_2_O_2_ presented significantly increased production of σ^B^-dependent alkaline shock protein 23 (*asp23)* than planktonic cells [137]. Also, genomic analyses demonstrated that the absence of thiol metabolic recycling genes and disruption of redox homeostasis in NTHi biofilms impact their survival under H_2_O_2_ stress [159]. Gene expression analysis of *S.* enteritidis biofilms treated with H_2_O_2_ and benzalkonium chloride (BAC) showed increased *invA* and *avrA* gene expression, which are linked to cellular invasion and anti-inflammatory response, respectively. This suggests that biofilm cells have enhanced invasion ability, potentially aiding pathogen survival in hosts [160]. The expression of *avrA* was also observed in *S. enterica* biofilms exposed to BAC and H_2_O_2_ [161].

### 3.6 Other players in the fight against oxidative stress in biofilms

As the knowledge of biofilm oxidative stress response advances, many mechanisms are unraveled (Table 4). Among them, the DNA damage response (DDR) is induced in mature *B. subtilis* biofilms as a response to increased levels of ROS and was shown to negatively affect the production of matrix genes [162]. In *P. aeruginosa* biofilms, the expression of *nrdJ*, a ribonucleotide reductase (RNRs) with a DNA-repairing effect, was induced by H_2_O_2_ [163]. In *P. aeruginosa* biofilms, the inactivation of the stringent response (SR), another mechanism employed by bacterial species against stress conditions, provoked an increase in the levels of hydroxyl radicals and the susceptibility of biofilms to paraquat and phenazine methosulfate due to the decreased activity of catalases and SOD [164]. Van Der Veen and Abee investigated the roles of HrcA, regulator of the class I heat-shock response, and the chaperone DnaK and found that these proteins are also involved in the protection of *L. monocytogenes* biofilms to benzalkonium chloride and peracetic acid [165].

**Table 4:** Summary of other genes involved in the oxidative stress resistance of bacterial biofilms to ROS and RCS.

Gene/System	Function	Bacterial species	Oxidizing agent	Reference
*nrdJ*	DNA repair	*P. aeruginosa*	H_2_O_2_	[163]
HrcA	Heat-shock response protein	*L. monocytogenes*	benzalkonium chloride	[165]
DnaK	Chaperone	*L. monocytogenes*	benzalkonium chloride	[165]
OxyR	Oxidative stress response regulator	*P. aeruginosa*	H_2_O_2_	[166]
OprL	Structural protein	*P. aeruginosa*	H_2_O_2_	[166]
BrlR	MerR transcriptional regulator	*P. aeruginosa*	H_2_O_2_	[167]
*gacA* and *gacS*	Virulence, metabolism, and biofilm formation	*Pseudomonas* species	H_2_O_2_	[168]

Among the transcriptional regulators, it was shown that, in addition to presenting increased susceptibility to H_2_O_2_, *P. aeruginosa oxyR oprL* double mutant biofilms presented alterations in the cell structure after H_2_O_2_ treatment compared to the *oxyR* mutant strain, highlighting the importance of oxidative stress response (OxyR) and the structural protein OprL in the resistance of biofilms to oxidizing agents [166]. Furthermore, the inactivation of *P. aeruginosa* PAO1 *brlR,* a member of the MerR family of transcriptional regulators, rendered continuous flow biofilms significantly more susceptible to H_2_O_2_ in a biofilm-specific way, while the expression of *brlR* in the Δ*brlR* mutant restored its susceptibility [167].

Endogenous H_2_O_2_ was shown to play an important role in the emergence of biofilm-derived *S. pneumoniae* small-colony variants [169]. In accordance, Davies et al. discovered that certain *Pseudomonas* species are prone to spontaneous mutations in *gacA* and *gacS*, in which *gacS* mutant biofilms generated phenotypically stable small colony variants, which exhibited resistance to H_2_O_2_ killing [168]. Moreover, exposure of *S. aureus* biofilms to H_2_O_2_ resulted in a higher biofilm mutability, and the addition of catalase reverted this phenotype, indicating that increased oxidative stress underlies the heightened mutability observed [170]. Research on *P. aeruginosa* biofilms revealed that oxidative stress induces double-stranded DNA breaks, leading to genetic variants through mutagenic repair mechanisms and biofilm diversity, ultimately aiding adaptation to changing environments and increasing antibiotic resistance, crucial for survival in harsh conditions [171].

## 4 Conclusions

**Fig. 3:**
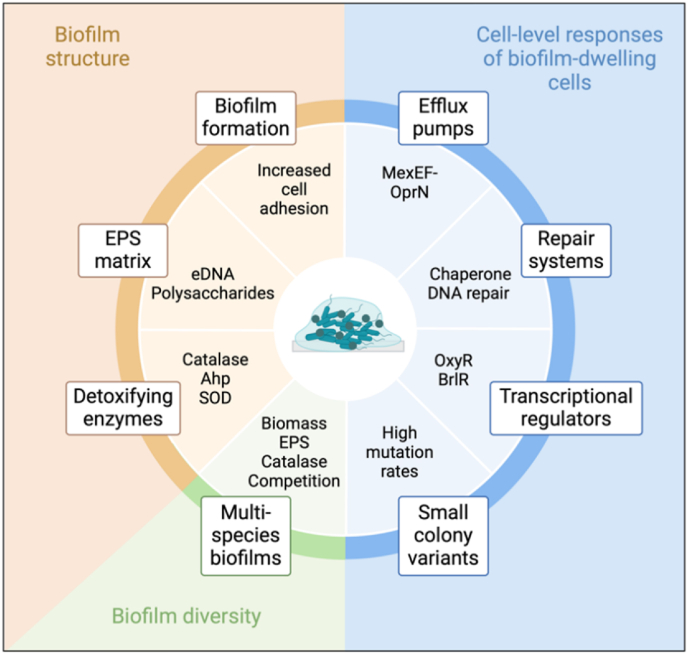
Summary of the oxidative stress response mechanisms employed by biofilms against ROS and RCS.

Understanding biofilm oxidative resistance mechanisms is a complex and ongoing area of research due to the diverse nature of biofilms and the multitude of factors that contribute to their resilience. Fig. 3 displays an overview of the mechanisms employed by biofilms against ROS and RCS. This review highlights the stimulation of biofilm formation, mainly during the initial steps of biofilm development, as an important protection mechanism against these toxic molecules since biofilms act as a shield for the cells. Furthermore, the EPS matrix, widely known for its protection against antibiotic treatment, also plays essential roles in biofilm resistance to ROS and RCS by interacting and quenching the toxic effect of these molecules. In this context, the reaction of EPS matrix components with oxidants would reduce the amount of toxic molecules reaching the cells, providing enough time for the activation or acquisition of other resistance traits. Detoxifying enzymes are well-known as essential molecules for the survival of planktonic cells to oxidative stress, and growing evidence indicates they are also employed by biofilms to inactive toxic reactive species. However, most biofilm studies have focused on H_2_O_2,_ and there is still a lack of information on the mechanisms behind the action of these enzymes on other oxidants, such as hypobromous acid, NaOCl and its active ingredient, HOCl, chloramines, and other widely used forms of ROS including peracetic acid, potassium monopersulfate, and ozone. In this regard, there seems to be a correlation between the defensive function of the EPS matrix and the activity of detoxifying enzymes. Additionally, growing as multispecies biofilms, as well as biofilm diversity and composition, also influence, either negatively or positively, the resistance of the biofilm-dwelling cells against ROS and RCS.

Although considerable effort has been made to study biofilms and their resistance mechanisms, most studies focus on antibiotics, and only a small proportion of them investigate the nature of biofilms' resistance to oxidants. In this context, some of the approaches that can be used to elucidate biofilm resistance mechanisms against the widely available oxidizing agents include Confocal Laser Scanning Microscopy (CLSM) and Atomic Force Microscopy (AFM) [172] to analyze biofilm population, spatial and temporal biofilm heterogeneity. RNA sequencing analyses using specific biofilm regions [173] or microscopy [172] can identify signaling pathways and molecules influencing biofilm formation and transcriptional heterogeneity. Characterization of the EPS matrix and the biochemistry behind its reaction with ROS and RCS can be measured by spectroscopy methods [174]. Oxidative damage can be quantified by measurement of lipid peroxidation by fluorometric methods, protein oxidation by liquid chromatography-mass spectrometry (LC-MS), and nucleic acid damage by ultra-performance LC-MS. Intracellular ROS can also be quantified by fluorescent probes like dichloro-dihydro-fluorescein diacetate (DCFH-DA) [175]. Moreover, microelectrode measurements can be used to detect spatial gradients of oxygen, pH, and other chemical parameters within biofilms, providing insights into microenvironmental conditions and metabolic activities at different depths and locations within the biofilm [172,176].

## Funding source

This work was supported by the Natural Sciences and Engineering Research Council of Canada (NSERC) under Discovery Grant [RGPIN-2019-06335], the Ontario Graduate Scholarship (OGS) program, and 10.13039/100008095Carleton University.

## CRediT authorship contribution statement

**Waleska Stephanie da Cruz Nizer:** Writing – review & editing, Writing – original draft, Investigation, Formal analysis, Conceptualization. **Madison Elisabeth Adams:** Writing – original draft, Investigation. **Kira Noelle Allison:** Writing – original draft, Investigation. **Megan Catherine Montgomery:** Writing – original draft. **Hailey Mosher:** Writing – original draft. **Edana Cassol:** Writing – review & editing, Supervision, Resources, Funding acquisition. **Joerg Overhage:** Writing – review & editing, Writing – original draft, Supervision, Resources, Funding acquisition, Formal analysis, Conceptualization.

## Declaration of competing interest

The authors declare that they have no known competing financial interests or personal relationships that could have appeared to influence the work reported in this paper.

## Data Availability

No data was used for the research described in the article.
